# T‐cell‐derived Hodgkin lymphoma has motility characteristics intermediate between Hodgkin and anaplastic large cell lymphoma

**DOI:** 10.1111/jcmm.17389

**Published:** 2022-05-19

**Authors:** Julia Bein, Nadine Flinner, Björn Häupl, Aastha Mathur, Olga Schneider, Marwa Abu‐Ayyad, Martin‐Leo Hansmann, Matthieu Piel, Thomas Oellerich, Sylvia Hartmann

**Affiliations:** ^1^ Dr. Senckenberg Institute of Pathology Goethe University Frankfurt am Main Frankfurt am Main Germany; ^2^ Frankfurt Cancer Institute Goethe University Frankfurt am Main Germany; ^3^ University Cancer Center (UCT) Frankfurt University Hospital Goethe University Frankfurt am Main Germany; ^4^ Frankfurt Institute for Advanced Studies Frankfurt am Main Germany; ^5^ Department of Internal Medicine 2 Goethe University Hospital Frankfurt Germany; ^6^ German Cancer Consortium/German Cancer Research Center Heidelberg Germany; ^7^ Institut Curie and Institut Pierre Gilles de Gennes PSL Research University CNRS UMR 144 Paris France; ^8^ Institute of General Pharmacology and Toxicology Goethe University Frankfurt am Main Frankfurt am Main Germany

**Keywords:** anaplastic large cell lymphoma, Hodgkin lymphoma, motility

## Abstract

Classic Hodgkin lymphoma (cHL) is usually characterized by a low tumour cell content, derived from crippled germinal centre B cells. Rare cases have been described in which the tumour cells show clonal T‐cell receptor rearrangements. From a clinicopathological perspective, it is unclear if these cases should be classified as cHL or anaplastic large T‐cell lymphoma (ALCL). Since we recently observed differences in the motility of ALCL and cHL tumour cells, here, we aimed to obtain a better understanding of T‐cell‐derived cHL by investigating their global proteomic profiles and their motility. In a proteomics analysis, when only motility‐associated proteins were regarded, T‐cell‐derived cHL cell lines showed the highest similarity to ALK^−^ ALCL cell lines. In contrast, T‐cell‐derived cHL cell lines presented a very low overall motility, similar to that observed in conventional cHL. Whereas all ALCL cell lines, as well as T‐cell‐derived cHL, predominantly presented an amoeboid migration pattern with uropod at the rear, conventional cHL never presented with uropods. The migration of ALCL cell lines was strongly impaired upon application of different inhibitors. This effect was less pronounced in cHL cell lines and almost invisible in T‐cell‐derived cHL. In summary, our cell line‐derived data suggest that based on proteomics and migration behaviour, T‐cell‐derived cHL is a neoplasm that shares features with both cHL and ALCL and is not an ALCL with low tumour cell content. Complementary clinical studies on this lymphoma are warranted.

## 1 INTRODUCTION

Classic Hodgkin lymphoma (cHL) and anaplastic large cell lymphoma (ALCL) both represent lymphomas with CD30+ tumour cells.^1^ cHL is frequently diagnosed in early stages of the disease,^2^ whereas ALCL is more frequently encountered with advanced‐stage disease and has a less favourable outcome.^3^ Despite the fact that these lymphomas originate from fundamentally different cells, with a B‐cell origin for cHL^4^ and a T‐cell origin for ALCL,^5^ both lymphomas have several features in common: both have lost expression of lineage‐specific surface markers and both have constitutively active NFKappaB, NOTCH1, AP1 and JAK‐STAT signalling.^6^, ^7^ ALCL is classified into ALK^+^ ALCL, with a rearrangement of the ALK gene, and ALK^−^ ALCL, which can harbour rearrangements of *DUSP22* or *TP63*.^8^, ^9^ For ALK^+^ ALCL, a Hodgkin‐like subtype is described, which is particularly difficult to differentiate from cHL purely by morphology.^10^ In this case, ALK1 protein expression is a helpful diagnostic tool. However, ALK^−^ ALCL can also present with a low tumour burden and may thus be difficult to distinguish from cHL since ALK1 is not expressed by ALK^−^ ALCL. Usually, demonstration of nuclear PAX5 staining is very helpful in this setting, as PAX5 is usually expressed in cHL but not in ALCL.^11^ However, the classification of some cases that do not express PAX5 remains unclear. PAX5^−^ cHL cases were shown to have inferior survival compared with conventional cHL cases.^12^ Demonstration of clonal B‐ or T‐cell receptor (TCR) rearrangements can be helpful in some cases, but due to the low tumour cell burden and extensive somatic hypermutation of immunoglobulin genes in cHL, clonal rearrangements are frequently missed. Immunohistochemical staining may provide some help in the diagnosis.^13^ In a subset of cases, an association with cutaneous ALCL has been observed, with single, scattered, large CD30^+^ tumour cells in the lymph nodes mimicking cHL.^14^ These cases represent a nodal manifestation of a cutaneous ALCL. However, few cases remain, in which no link to a cutaneous T‐cell lymphoma can be demonstrated. Based on single‐cell polymerase chain reaction (PCR), clonal rearrangements of the TCR could be demonstrated in such cases.^15^ However, even two of the recognized cHL cell lines, namely L‐540 and HDLM‐2, harbour clonal TCR rearrangements, thus originating from T cells. HDLM‐2 was established from the pleural effusion of a 74‐year‐old man with Hodgkin lymphoma (nodular sclerosing; stage IV) in 1982.^16^ L‐540 was established from the bone marrow of a 20‐year‐old woman with Hodgkin lymphoma (nodular sclerosing; stage IVB).^17^ In gene expression studies, these cell lines clustered very well with cHL.^18^ Thus, the biology of cases with the morphology of cHL and clonal TCR rearrangement in the tumour cells is still unclear. The typical morphology and immunophenotype of the tumour cells, the microenvironment, and the crippled B‐cell receptor genes are all part of the definition of cHL. However, in these particular cases with cHL morphology and clonal TCR rearrangement in the neoplastic cells, traditional classification criteria do not help any further. As we recently observed that malignant lymphomas differ in their migratory properties,^19^ the present study was based on the idea that an analysis of the motility of such tumour cells could help to better understand and classify these lymphomas. Therefore, the present study aimed to better understand the biology and classification of T‐cell‐derived cHL neoplasms with a focus on the T‐cell‐derived cHL cell lines L‐540 and HDLM‐2 and their migratory behaviour.

## 2 METHODS

### 2.1 Proteomics

The cell lines L‐428 (RRID:CVCL_1361), L‐1236 (RRID:CVCL_2096), DEL (RRID:CVCL_1170), SU‐DHL‐1 (CVCL_0538), HDLM‐2 (RRID:CVCL_0009) and L‐540 (RRID:CVCL_1362) were purchased from the German Collection of Microorganisms and Cell Cultures (Braunschweig, Germany). The cell lines Mac‐1 (RRID:CVCL_H631) and Mac‐2A (RRID:CVCL_H637) were provided by Prof. Olaf Merkel, Medical University of Vienna, Austria. The identity of all cell lines was authenticated by short tandem repeats (STR) profiling. All cell lines were regularly tested for mycoplasma contamination.

All cell lines underwent quantitative proteomics based on tandem mass tagging (TMT). For this, 5 × 10^5^ cells were harvested and lysed in urea buffer as previously described.^20^

After protein extraction and digestion with trypsin, peptides were labelled with TMT 10‐plex reagents (Thermo Fisher Scientific, Dreieich, Germany). The individually labelled peptides were combined in multiplexed samples. For normalization among multiplexes, an internal reference consisting of peptides from each condition was included. After pre‐fractionation using a high‐pH C18 reversed‐phase kit (Thermo Fisher Scientific), the TMT‐labelled peptide mixtures were separated by C18‐reversed‐phase‐HPLC on an Ultimate 3000 RSLCnano system (Thermo Fisher Scientific). Peptides eluted from the analytical column were analysed by nano‐ESI Orbitrap tandem mass spectrometry on a Q Exactive HF instrument (Thermo Fisher Scientific). The raw mass spectrometric data were processed with the MaxQuant software (version 1.6.17.0; Max Planck Institute of Biochemistry [MPIB]).^21^ This included the identification of proteins by running the mass spectra against the Uniprot human protein database (downloaded in February 2019) and collection of common laboratory contaminants, followed by the extraction of TMT reporter ion intensities for protein quantitation. After recalibration, the mass tolerances for precursor and fragment ions were set to 4.5 and 20 ppm, respectively. Oxidation of methionine and acetylation of the protein *N*‐terminus were considered variable modifications, and carbamidomethylation of cysteine was defined as a fixed modification. The minimal peptide length was set to seven amino acids, allowing up to two missed tryptic cleavages. Both at the peptide and protein levels, a maximum false discovery rate (FDR) of 1% was applied using a reversed decoy database. For downstream data processing with the Perseus software (version 1.6.0.7; MPIB), potential contaminants, proteins identified solely with modified peptides, and hits in the decoy database were removed. After normalization of TMT reporter ion intensities across multiplexed samples, the data were subjected to unsupervised hierarchical clustering based on the Euclidean distance and average linkage method, either using all quantified protein groups or after mapping motility‐related proteins. The mass spectrometry proteomics data have been deposited to the ProteomeXchange Consortium via the PRIDE^22^ partner repository with the dataset identifier PXD031907 (https://www.ebi.ac.uk/pride).

### 2.2 Microchannel experiments

Polydimethylsiloxane (PDMS) chips with different types of microchannels were produced in moulds provided by Dr. Matthieu Piel. Straight channels with a diameter of 8 µm and height of 10 µm were tested and found to be most appropriate for the rather large Hodgkin and Reed‐Sternberg (HRS) tumour cells of cHL cell lines. For constriction experiments, channels with 12 µm × 12 µm shape and 12 µm × 4 µm constriction were chosen. Microchannels were coated with fibronectin for at least 1 h. The inhibitors Y27632 (Meck, Darmstadt, Germany), blebbistatin and CK‐666 (both Cayman Chemicals, Ann Arbor, MI, USA) were applied in specified doses before the cells were loaded into the chips. Prior to the experiments, the optimal inhibitor doses were determined by a titration curve, identifying dosages that had only minor effects on cell viability (data not shown). 10^5^ cells with or without inhibitors were loaded into the PDMS chip, and the chip was pre‐incubated for 1 h in the incubator. Cell motility was monitored using a phase‐contrast microscope under incubator conditions for 20 h, with time‐lapse images taken every 10 min in straight channels and every 4 min in microchannels with constrictions. Cells in videos from microchannel experiments were segmented with a custom script (available on request; see below for a description of the procedure). The X and Y positions of the segmented cells were used as input for tracking, which was done with TrackMate^23^ using the LAP tracker (parameters: maximum distance for frame‐to‐frame linking: 100; maximum distance for gap closing: 150; maximal gap length: 15; penalty for linking cells with different Y positions: Y15). The step‐based velocity and straightness were calculated based on the X, Y and T coordinates of the tracks. Cell movements in microchannels with constrictions were manually assessed (the number of cells passing a particular constriction was counted and the time required for the passage of the constriction was calculated according to the number of image frames that were taken until the passage was complete). For some exemplary experiments, Hoechst 33342 (ab228551, Abcam, Cambridge, UK) was added to the cell culture medium in order to highlight nuclei. The study was approved by the Institutional Review Board of the University Hospital Frankfurt (No. 20‐876).

### 2.3 Segmentation of movies from microchannel experiments

Segmentation was done with the scikit‐image package in Python.^24^ First, noise was removed with a median filter, and a background image was calculated by taking the median pixel value for each position over all frames of the video. Then, this background image was subtracted from each frame to remove the microchannels from the image. A mean filter was applied to smoothen the image again, and a black top‐head filter was used to enhance the signal of the cells. Finally, the cells were detected with a Yen threshold, and morphological operations were used to improve the result.

### 2.4 Immunofluorescence

Cytospins from all cell lines were prepared and incubated with a monoclonal anti‐lamin A/C antibody (1:200; sc‐7292, Santa Cruz Biotechnologies, Dallas, USA). The VectaFluor Excel Antibody Kit Dylight 594 (Vector Laboratories, Burlingame, CA, USA) was used for detection. A Leica TCS SP8 confocal microscope (Leica Microsystems, Wetzlar, Germany) was used for 3D imaging. The settings were as follows: HC PL APO 63x/1.3 GLYC CORR, Cs2; lasers: 405 nm DMOD Compact, Red 594 nm and Green 488 nm. The presented pixel size was 130 nm in each coordinate direction. Furthermore, the z‐step had a size of 0.13 mm.^25^, ^26^

## 3 RESULTS

### 3.1 Global proteomic profiles show similarities in the expression of motility‐related proteins between T‐cell‐derived cHL cell lines and ALK^−^ ALCL

**FIGURE 1:**
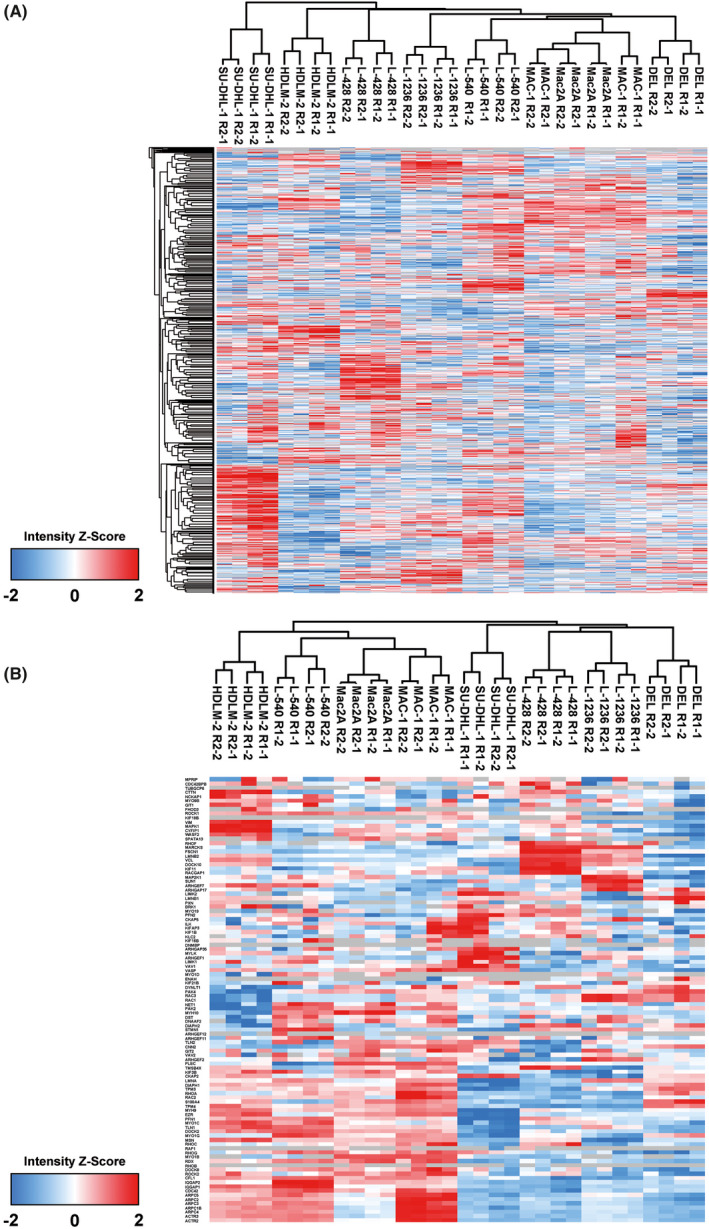
Unsupervised hierarchical clusters of ALCL and Hodgkin cell lines according to proteomics data. (A) Unsupervised hierarchical clustering based on all quantified protein groups. (B) Unsupervised hierarchical clustering after mapping of proteins related to cell movement

Since we were interested in seeing if the two T‐cell‐derived cHL cell lines had proteomic profiles that were more similar to those of B‐cell‐derived cHL or ALCL, global proteomic profiles were determined and compared. Both the T‐cell‐derived cHL cell lines, L‐540 and HDLM‐2, clustered together with the cHL cell lines L‐428 and L‐1236 in the global unsupervised cluster (Figure 1A). However, in an unsupervised cluster including only motility‐related genes, L‐540 and HDLM‐2 clustered closely with the ALK^−^ ALCL cell lines MAC‐1 and MAC‐2A, indicating that the intracellular machinery applied for cell movements is more similar to that of ALK^−^ ALCL (Figure 1B). Subunits of the actin‐related protein (Arp)2/3 complex were expressed at 1.33‐fold to 1.59‐fold higher levels in the T‐cell‐derived cHL cell lines L‐540 and HDLM‐2 compared with the cHL cell lines L‐428 and L‐1236. MYH9, a component of the myosin II complex, also showed 1.46‐fold higher expression in L‐540 and HDLM‐2 compared with L‐428 and L‐1236. CDC42 showed 1.63‐fold higher expression in L‐540 and HDLM‐2 than in L‐428 and L‐1236.

### 3.2 T‐cell‐derived cHL cell lines share baseline movement characteristics with both cHL and ALK^−^ ALCL cell lines

**FIGURE 2:**
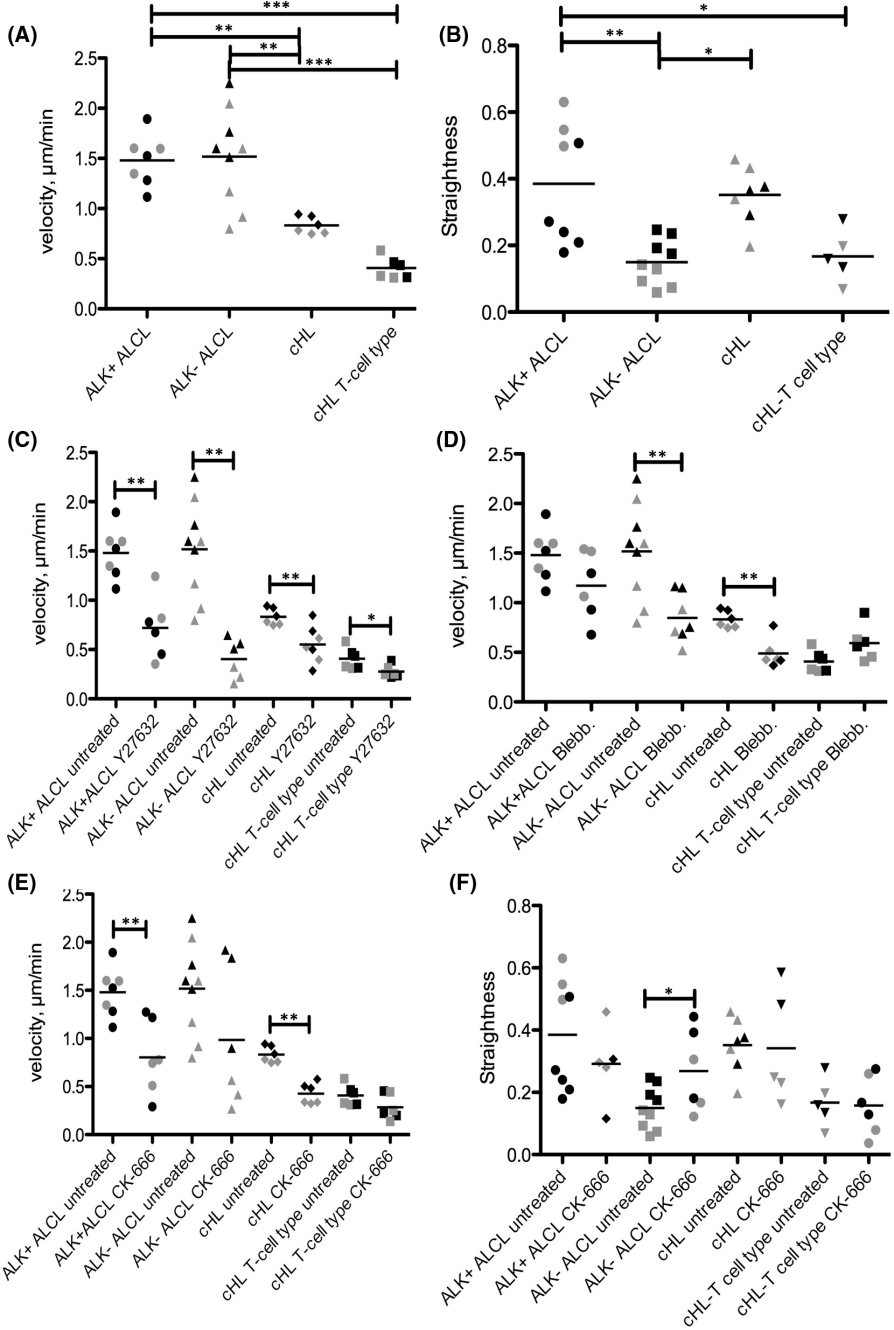
Baseline movement characteristics of ALCL and Hodgkin cell lines and movement after inhibitor treatment in straight microchannels with height 10 µm and width 8 µm. (A) Step based velocity of ALK^+^, ALK^−^ ALCL and Hodgkin cell lines. At least three independent experiments per cell line (***p < 0.001, **p < 0.01, one‐way ANOVA with Bonferroni´s post‐test for multiple comparisons). (B) Straightness of ALK^+^, ALK^−^ ALCL and Hodgkin cell lines. At least three independent experiments per cell line (***p < 0.001, **p < 0.01, one‐way ANOVA with Bonferroni´s post‐test for multiple comparisons). (C). Step based velocity of ALK^+^, ALK^−^ ALCL and Hodgkin cell lines after treatment with the ROCK inhibitor Y27632 (30 µM). At least three independent experiments per cell line (*p < 0.05, **p < 0.01, Mann–Whitney test). (D) Step based velocity of ALK^+^, ALK^−^ ALCL and Hodgkin cell lines after treatment with the myosin II inhibitor Blebbistatin (15 µM). At least three independent experiments per cell line (**p < 0.01, Mann–Whitney test). (E) Step based velocity of ALK^+^, ALK^−^ ALCL and Hodgkin cell lines after treatment with the actin inhibitor CK‐666 (50 µM). At least three independent experiments per cell line (**p < 0.01, Mann–Whitney test). (F) Straightness of ALK^+^, ALK^−^ ALCL and Hodgkin cell lines after treatment with the actin inhibitor CK‐666 (50 µM). At least three independent experiments per cell line (*p < 0.05, Mann–Whitney test). Labelled in grey: ALK^+^ ALCL: DEL, ALK^−^ ALCL: MAC2A, cHL: L‐1236, T‐cell‐derived cHL: L‐540. Labelled in black: ALK^+^ ALCL: SU‐DHL‐1, ALK^−^ ALCL: MAC1, cHL: L‐428, T‐cell‐derived cHL: HDLM‐2

Since we previously observed differences in cell migration between ALCL and cHL,^27^ we were now interested in studying how T‐cell‐derived cHL cell lines move. Looking at the baseline migration velocity, we could confirm in microchannels with a width of 8 µm and a height of 10 µm that the cHL cell lines L‐1236 and L‐428 move at a significantly lower velocity (0.76 and 0.90 µm/min, respectively) than ALK^+^ (1.52 and 1.45 µm/min) and ALK^−^ ALCL cell lines (1.63 and 1.31 µm/min; one‐way ANOVA with Bonferroni´s post‐test for multiple comparisons; Figure 2). The T‐cell‐derived cHL cell lines L‐540 and HDLM‐2 were even slower (both 0.41 µm/min) compared with the cHL cell lines L‐1236 and L‐428; however, this difference was not significant.

We also analysed the straightness of cell movements, defined as the absolute track length of a cell relative to the displacement of that cell. Whereas the straightness in the movement of ALK^+^ ALCL cell lines was heterogeneous, with high straightness in SU‐DHL‐1 cells (0.34) and lower straightness in DEL cells (0.19), ALK^−^ ALCL and T‐cell‐derived cHL cell lines both had significantly lower straightness (0.20 and 0.10 in ALK^−^ ALCL vs 0.19 and 0.13 in T‐cell‐derived cHL cells; one‐way ANOVA with Bonferroni´s post‐test for multiple comparisons; Figure 2A), which means that the cells frequently turned forward and backward, a pattern that may resemble the antigen‐scanning activity of normal T cells. In contrast, both the cHL cell lines, L‐428 and L‐1236, displayed relatively high straightness (0.34 and 0.33).

Regarding the morphology of moving cells, the cHL cell lines L‐1236 and L‐428 crawled forward slowly, resembling the previously described A1 mode of amoeboid migration.^28^ Cells of the T‐cell‐derived cHL cell lines predominantly presented with very little movement, with a few cells having a high velocity and a uropod‐like structure at the rear end of the cell as in the previously described A2 mode of amoeboid migration. The A2 mode was never observed in the cHL cell lines L‐428 and L‐1236. In contrast, the ALK^+^ ALCL cell lines DEL and SU‐DHL‐1 frequently presented fast cells having A2 morphology. The ALK^−^ ALCL cell lines MAC1 and MAC2A showed high plasticity between the different types of migration, with some cells presenting a mesenchymal morphology while others displayed A1 and A2 morphology. In particular, MAC2A had frequently turning cells that flipped backward and forward (Figure 2B, Supporting Information Movies S1–S8).

### 3.3 Inhibitors impair the cells’ velocity but have little impact on the mode of migration

**FIGURE 3:**
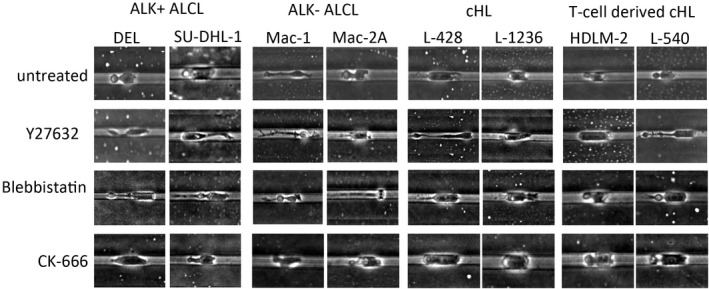
Morphology of moving cells at baseline and after inhibitor treatment in straight microchannels with height 10 µm and width 8 µm. The ALK^+^ ALCL cell lines DEL and SU‐DHL‐1, ALK^−^ ALCL cell lines MAC1 and MAC2A, cHL cell lines L‐428 and L‐1236 and T‐cell‐derived cHL cell lines L‐540 and HDLM‐2 are displayed. First line, cells are shown at baseline, second line after ROCK inhibition with 30 µM Y27632, third line after myosin II inhibition with 15 µM Blebbistatin, last line after actin‐related protein Arp2/3 complex inhibition with 50 µM CK‐666 is shown. Representative examples in phase‐contrast microscopy at 10x magnification were chosen

Next, we aimed to decipher which migration‐related factors have major effects on the motility of ALCL and cHL tumour cells. First, we applied the ROCK inhibitor Y27632 (30 µM) and analysed the effect on cells’ ability to migrate. All the tested cell lines showed a significant impairment of their motility after the application of Y27632 (Mann–Whitney test), with a complete disappearance of the A2 mode of movement. The effect was strongest in the ALK^+^ and ALK^−^ ALCL cell lines, particularly in the ALK^−^ ALCL cell line MAC2A, whereas the effects on the cHL and T‐cell‐derived cHL cell lines were less pronounced (Figure 2C). The morphology of migration was comparable in all treated cell lines, which showed mainly cells with A1 morphology and protrusions of the cell membrane, which occurred frequently at the rear of the cell but also at the leading edge of the cell in some cells (Table 1, Figure 3, Supporting Information Figures S1 and S2, and Movies S9–S11).

**TABLE 1:** Preferential mode of movement observed in moving cells according to Agarwal et al.^28^

Lymphoma	Cell Line	Baseline	Y27632	Blebbistatin	CK−666
ALK^+^ ALCL	DEL	A1 + A2	A1 + mesenchymal	A1 + A2 with protrusions at rear	A1 + A2, many cells stop
	SU‐DHL−1	A1 + A2	A1 with protrusions	A1 + A2 with protrusions at rear	A1 + A2, many cells stop
ALK^−^ ALCL	MAC1	A1 + A2	mesenchymal	A1 + A2 with protrusions at rear	A1 + A2
	MAC2A	A1 + A2	A1/no motility	A1 + A2 with protrusions at rear	A1 + A2
cHL	L−428	A1	A1 with protrusions	A1 with protrusions at rear	A1
	L−1236	A1	A1 with protrusions	A1 with protrusions at rear	A1
cHL T‐cell‐derived	HDLM−2	A1 + A2	A1	A1 + A2 + mesenchymal	A1 + A2
	L−540	A1 + A2	A1 with protrusions	A1 + A2 + mesenchymal	A1 + A2

Note

Movies were analysed for the morphology of moving cells. Immotile cells were neglected in this analysis. A1 and A2 represent forms of amoeboid movement.

Next, we tested the effect of the myosin II inhibitor blebbistatin (15 µM) on all cell lines. Significant differences in velocity were observed here between the ALK^−^ ALCL cell lines and cHL cell lines. Surprisingly, the T‐cell‐derived cHL cell lines could even move slightly better after blebbistatin application (Figure 2D), suggesting that these cell lines may still be able to move after myosin II inhibition in relation to their higher endogenous myosin II levels as observed in the global proteomics screening. After blebbistatin application, the morphology of the migrating cells changed regularly in that the cells had problems with the retraction of their rear, leading to long tails at their rear ends (Figure 3, Supporting Information Figure S3, and Movies S12–S14). However, cells moving in the A2 mode were occasionally encountered, sometimes with a long tail at the uropod (Figure 3, MAC1 and L‐540).

After inhibition of the Arp2/3 complex using the inhibitor CK‐666 (50 µM), all cell lines showed impaired cell motility, which was significant for the ALK^+^ ALCL cell lines and cHL. Whereas treatments with Y27632 and blebbistatin had no effects on the straightness of the cells, surprisingly, the ALK^−^ ALCL cell lines MAC1 and MAC2A presented a significant increase in straightness after CK‐666 administration, which was probably related to the low overall velocity, with a few cells having A2 morphology moving in a straight manner at a relatively high velocity. The morphology of the CK‐666‐treated cells was little changed compared with untreated cells (Figure 3).

### 3.4 ALK^+^ ALCL are superior to cHL at overcoming constrictions in microchannels due to more flexible nuclear laminae

**FIGURE 4:**
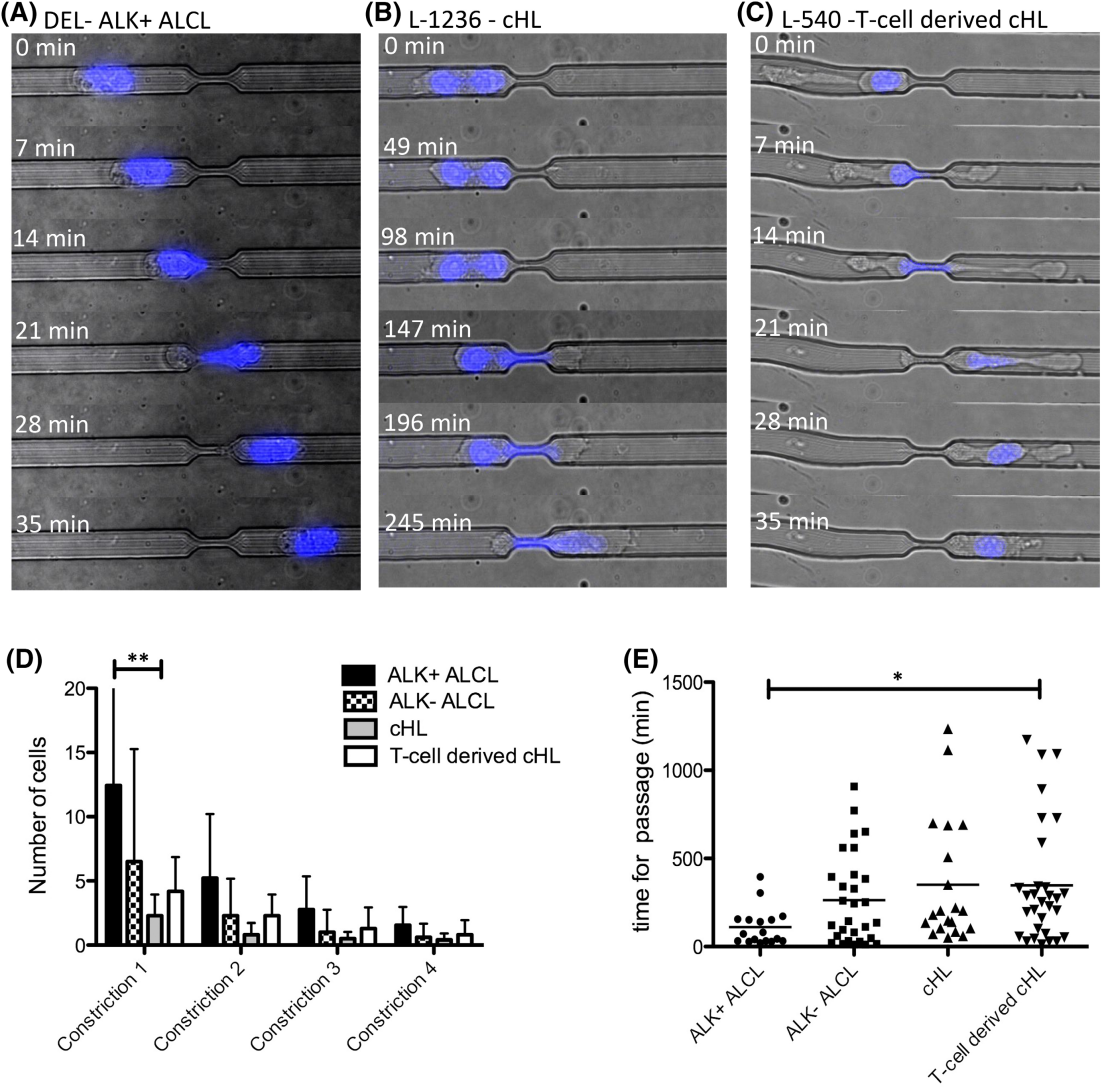
Movement of Hodgkin and ALCL cell lines in microchannels with 12 µm diameter and 4 µm constrictions. (A) Passage of the first constriction by a DEL cell (ALK^+^ ALCL) within 28 min. The nucleus is highlighted in blue (Hoechst dye), 40× magnification. (B) Passage of the first constriction by an L‐1236 cell (cHL) within 266 min. The nucleus is highlighted in blue (Hoechst dye), 40× magnification. (C) Passage of the first constriction by an L‐540 cell (T‐cell‐derived cHL) within 35 min. The nucleus is highlighted in blue (Hoechst dye), 40× magnification. (D) Number of cells passing the first four constrictions according to number of constriction and type of lymphoma. ** *p* < 0.01, Kruskal–Wallis test with Dunn´s post‐test for multiple comparisons. 4–5 replicates for each cell line, two cell lines per lymphoma entity. (E) Time (min) required for the passage of the first constriction. Each dot represents one cell. Two cell lines per lymphoma entity. *p < 0.01, Kruskal–Wallis test with Dunn's post‐test for multiple comparisons

Since we were also interested in elucidating how the different lymphoma cell lines would deal with severely confined spaces similar to the passage of tumour cells between endothelial cells, migration in microchannels with 12 × 4 µm‐wide constrictions was monitored. Whereas most of the ALK^+^ ALCL cells could pass through several constrictions with a relatively high velocity (Figure 4), cHL cell lines, including the T‐cell‐derived cHL cell lines, were less efficient at passing constrictions. Most cells of the cHL and T‐cell‐derived cHL cell lines spent a long time in front of or within the constriction compared with ALK^+^ ALCL cell lines. The time required to pass the first constriction was significantly higher for T‐cell‐derived cHL cell lines compared with ALK^+^ ALCL (*p* < 0.05, Kruskal–Wallis test with Dunn's post‐test for multiple comparisons; Figure 4). Again, this fact points to T‐cell‐derived cHL cell lines occupying an intermediate position between ALCL‐ and B‐cell‐derived cHL cell lines.

**FIGURE 5:**
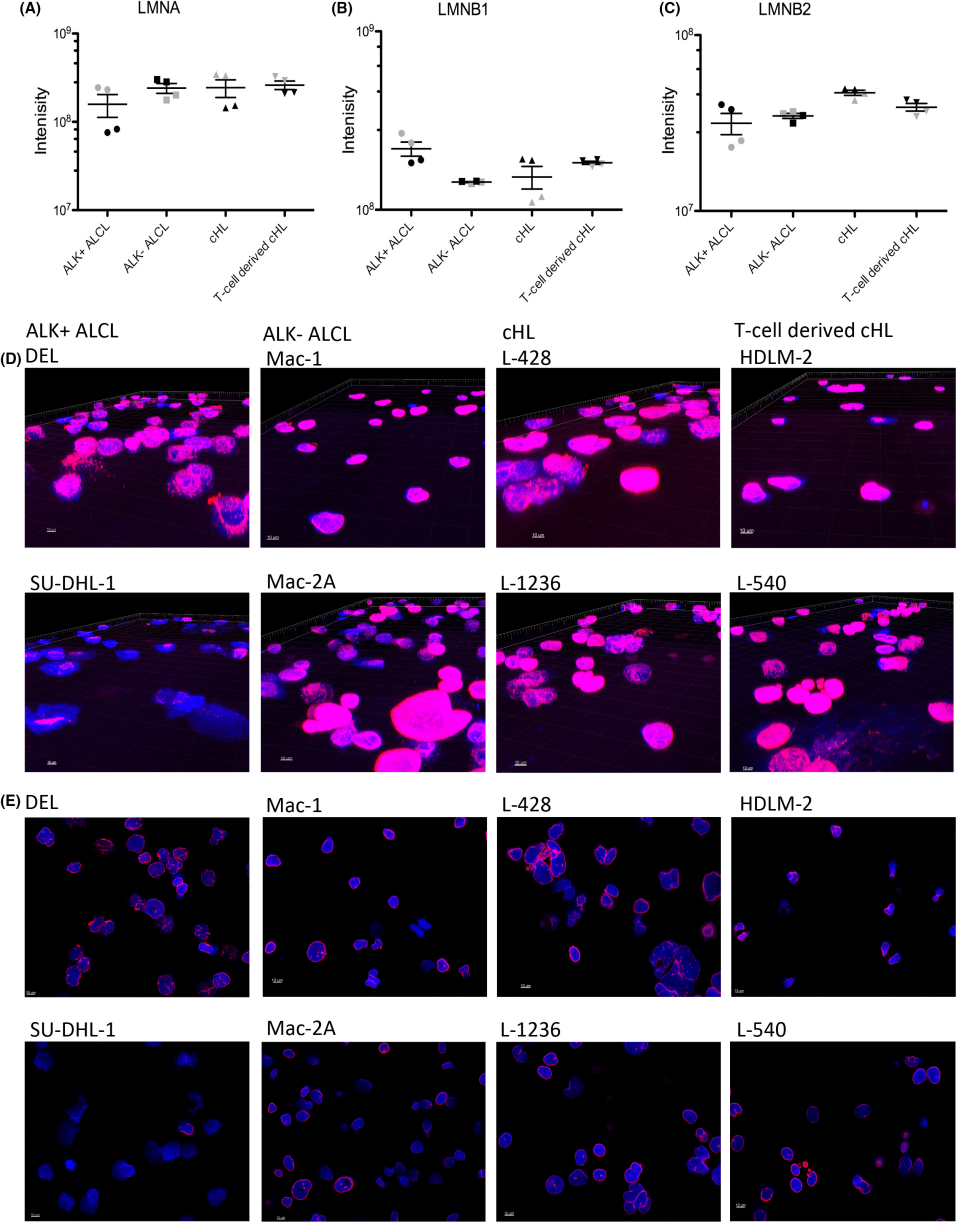
Lamin A/C expression in the nuclear laminae of Hodgkin and ALCL cell lines. (A) Lamin A protein expression in cHL and ALCL cell lines according to global proteomics data. (B) Lamin B1 protein expression in cHL and ALCL cell lines according to global proteomics data. (C) Lamin B2 protein expression in cHL and ALCL cell lines according to global proteomics data. Labelled in grey: ALK^+^ ALCL: DEL, ALK^−^ ALCL: MAC2A, cHL: L‐1236, T‐cell‐derived cHL: L‐540. Labelled in black: ALK^+^ ALCL: SU‐DHL‐1, ALK^−^ ALCL: MAC1, cHL: L‐428, T‐cell‐derived cHL: HDLM‐2. (D) 3D confocal image of cell nuclei in Lamin A/C immunofluorescence, nuclei counterstained with DAPI, 63× magnification, cell lines as indicated. (F) Cut section through cell nuclei in Lamin A/C immunofluorescence, nuclei counterstained with DAPI, 63× magnification, cell lines as indicated

Passage through constrictions can be limited due to nuclear stiffness, wherein lamin A (and its splice variant lamin C) contributes to the stiffness of the nuclear lamina, while lamin B1 and B2 contribute to a more flexible nuclear lamina. Therefore, we checked proteomics data for lamin A and B1/B2 expression. ALK^+^ ALCL cell lines had the lowest lamin A protein expression (mean intensity value 158,257,750 in ALK^+^ ALCL vs mean intensity values 240,863,250, 244,062,500 and 260,886,750 in ALK^−^ ALCL, cHL and T‐cell‐derived cHL, respectively). In contrast, ALK^+^ ALCL had the highest lamin B1 expression (mean intensity value 219,675,000 vs mean intensity values 142,970,750, 152,759,000 and 183,119,500 in ALK^−^ ALCL, cHL and T‐cell‐derived cHL, respectively), suggesting relatively soft and flexible nuclei in ALK^+^ ALCL (Figure 5) compared with the other cell lines. All the tested cell lines were also investigated by immunofluorescence for lamin A/C protein expression. Here, we observed the strongest expression of lamin A/C in cHL and T‐cell‐derived cHL cell lines, with a somewhat more heterogeneous distribution of lamin A/C expression in ALK^−^ ALCL cell lines and a weak and heterogeneous expression in ALK^+^ ALCL cell lines (Figure 5). Thus, lamin A/C expression was inversely correlated with the ability of the cell lines to pass through constrictions.

## 4 DISCUSSION

To our knowledge, this study is the first to assess cell motility in order to classify different lymphoma cell lines. We could demonstrate in previous studies^19^, ^27^ that lymphoma entities with similarities in morphology, immunohistochemistry and molecular characteristics show differences in their migratory properties. Although cHL with clonal TCR rearrangements have been described,^15^ due to the rarity of such cases, their existence was doubted and it was unclear how best to classify them. Here, we present data showing that the morphology of the moving cells from T‐cell‐derived cHL cell lines has similarities to ALCL, which probably predominantly relates to the T‐cell origin of the cells. However, the low ability of the T‐cell‐derived HRS cells to move at all bears a higher resemblance to cHL. The morphological hallmarks of HRS cells—huge, pleomorphic, sometimes binucleated—correlate well with the inability to move, which we could also demonstrate for cHL in primary tissue samples.^19^ The inability to move of T‐cell‐ and B‐cell‐derived cHL cell lines matches with the clinical observation that primary PAX5^−^ cHL cases expressing cytotoxic molecules (and thus, probably T‐cell‐derived) did not differ in stage or LDH from other cHL cases,^12^ whereas peripheral T‐cell lymphomas presented with advanced‐stage disease significantly more frequently. The inability of HRS cells to propagate from one lymph node to the other is reflected in the frequent limited‐stage disease and contiguous lymph node involvement.

The rarity of these lymphoma cases was also the reason we did not have the chance to study suspensions of such primary cases in microchannels. Although we tested suspensions of conventional primary cHL and ALCL, the number of CD30+ tumour cells entering microchannels was too low to draw any meaningful conclusions. The majority of moving cells here represented reactive T cells from the microenvironment. Therefore, we must currently rely on T‐cell‐derived cHL cell lines as a good model for this disease.

T‐cell‐derived cHL cell lines presented some moving cells with a uropod and typical amoeboid A2 morphology, which we also observed in ALCL cell lines. Formation of an uropod with clustering of TCRs in a dynamic immunological synapse, also called kinapse,^29^ is frequently observed for normal T cells.^30^ Therefore, the manner of movement of T‐cell‐derived cHL cell lines reflects their T‐cell origin. However, the generally strongly impaired motility in T‐cell‐derived cHL cell lines resembles B‐cell‐derived cHL.

With regard to susceptibility towards different inhibitors, the ROCK inhibitor Y27632 had some effect on the T‐cell‐derived cHL cell lines. As observed in almost all cell lines, long tails at the rear of the cell were seen, as also previously described in the context of monocyte tail retraction after *Rhoa* blocking.^31^ Whereas ALCL cell lines presented a strongly decreased motility after the application of most inhibitors, the effects were less pronounced in cHL cell lines and almost abrogated in T‐cell‐derived cHL cell lines, indicating that T‐cell‐derived cHL cells already have impaired movement machinery at baseline. For ALK^+^ ALCL, the presence of the NPM‐ALK fusion gene has been described to positively regulate the movement of the cells by phosphorylation of VAV1 and VAV3 and consequent activation of RAC1.^32^, ^33^ Additionally, it was shown that the NPM‐ALK fusion gene contributes to the polarization of the cells and replaces stimulation of the TCR. However, in our data, ALK^+^ ALCL cell lines behaved similarly to ALK^−^ ALCL in their migration, although the activated cytoskeleton components may differ, as we also observed through the global proteomics approach. Both ALK^+^ and ALK^−^ ALCL cell lines were efficient in passing 4‐µm constrictions, particularly for the first constriction in the series. The mechanics and stiffness of the nuclear membrane of the lymphoma cells may lead to nuclear rupture and DNA damage during passage through a constriction,^34^, ^35^ resulting in apoptosis. In line with the inability of cHL cell lines to pass through constrictions, we observed strong expression of lamin A/C in the nuclear lamina. Increased segmentation of the nuclei by lamin A/C has previously been observed in tumour cells of cHL.^36^

Based on the low efficiency of movement, T‐cell‐derived cHL cell lines are different from other CD30+ T‐cell lymphoma cell lines. In gene expression profiling, T‐cell‐derived cHL cell lines closely resembled conventional cHL cell lines.^18^ However, the clinical outcome of PAX5^−^ cHL after cHL treatment was inferior to that of conventional cHL,^12^ suggesting that there are differences between B‐cell‐ and T‐cell‐derived cHL. Studies comparing the clinical behaviour of T‐cell‐derived cHL with conventional ALCL are lacking so far due to the rarity of these cases.

In summary, our cell line‐derived data suggest that cases with the morphology of cHL and T‐cell origin of the neoplastic cells (T‐cell‐derived cHL) should probably be merged neither with cHL nor with ALCL. An in‐depth characterization of the clinical behaviour of a larger number of such cases is warranted.

## AUTHOR CONTRIBUTION

**Julia Bein:** Data curation (lead); Formal analysis (equal); Investigation (equal). **Nadine Flinner:** Methodology (equal); Resources (equal); Software (lead). **Björn Häupl:** Data curation (equal); Formal analysis (equal); Visualization (equal). **Aastha Mathur:** Data curation (equal); Formal analysis (equal). **Olga Schneider:** Data curation (equal); Formal analysis (equal). **Marwa Abu‐Ayyad:** Software (equal). **Martin‐Leo Hansmann:** Conceptualization (equal); Supervision (equal). **Matthieu Piel:** Conceptualization (equal); Methodology (lead); Project administration (equal); Resources (equal); Supervision (equal). **Thomas Oellerich:** Data curation (equal); Resources (equal); Supervision (equal). **Sylvia Hartmann:** Conceptualization (lead); Data curation (equal); Formal analysis (equal); Funding acquisition (supporting); Investigation (equal); Project administration (equal); Supervision (lead); Visualization (lead); Writing – original draft (lead).

## CONFLICT OF INTEREST

The authors do not report any conflict of interest.

## Data Availability

The mass spectrometry proteomics data have been deposited to the ProteomeXchange Consortium via the PRIDE^22^ partner repository with the dataset identifier PXD031907 (https://www.ebi.ac.uk/pride). Raw data of movies are available on request from the corresponding author (several hundred Megabytes for each movie).
